# The Chronic Running Ear

**Published:** 1916-07

**Authors:** Ernest S. Colvin

**Affiliations:** Atlanta, Ga.


### THE CHRONIC RUNNING EAR.

By Earnest S. Colvin, M.D., Atlanta, Ga

The chronic running ear is simply a continuation of the acute running ear, the causes of which are those of catarrh in any other part of the body. We are all catarrh specialists together, in the sense that a great part of our work and much special attention is paid to the treatment of inflammations of mucous membranes in one part or another of the body. These catarrhs, so-called, consist of irritations or inflammation of the mucous membrane coming from the reaction between the defensive forces of nature and the pathogenic microbes. Of these, by far the most common are the following, probably in the order of their frequency: the pneumococcus, staphylococcus, streptococcus, bacillus influenzae and, to a less extent, bacillus diphtheriae, and many other pus forming microbes. The infections of scarlet fever and measles are also very common exciting causes.

Infection attacks the middle ear principally in one of two methods: First and most frequently, by extension upward from the throat through the Eustachian tube into the middle ear cavity. Second, and this distinction is clinical rather than pathological, in the more general infection, where the catarrhal trouble attacks not only the nose and throat, but the ear and many of the sinuses at the same time, as in the typical grippe, whether pneumococcic influenzae, streptococcic, staphylococcic, etc., in acute infetion diseases such as measles, scarlet fever, small-pox, and others.

Whenever an otitis runs into the chronic form, there must be one or more of three factors contributing to this. First, there may be continual local irritation, as the presence of diseased adenoids, post-nasal discharge, pressure from polypi or other tumors. Second: There may be, and probably is, a condition of poor drainage and ventilation, thus the Eustachian tube may have been nearly closed from former catarrhal thickening, the disease may have located in the attic of the middle ear, or may have extended not only to the middle ear proper, but to the antrum of mastoid and even the ultimate mastoid cells. Third: As in any other chronic condition in any other part of the body, there may be a decreased resisting power of the general system, notably as in tuberculosis, many forms of anemia, general weakness from run-down condition after sickness, or accompanying any form of systemic trouble.

The pathology of these catarrhal attacks need not be here gone into, merely calling attention to the fact that in the majority of the chronic catarrhs the germ at fault is usually found well below the surface of the membrane in the various mucous glands.

It is unnecessary here to mention the symptomatology of the chronic running ear. The presence of the continued or recurring discharge makes the diagnosis. When this discharge is examined with microscope it may be found to consist merely of mucus and pus cells where none of the infecting organisms are found, or these germs may be easily discovered in a smear. Much importance attaches to the germ that is causative in these cases. Along with the search for the exciting organism, a smear should be examined for the presence of bone detritus, as it is above all, necessary to know whether the infection has begun to destroy the bony tissue itself.

Many a case of otitis can be recognized as of chronic character from the very inception. Other cases so simulate the typical acute case that one has no means of recognizing on the start whether it will heal promptly or go into chronicity. The acute running ear, under proper treatment should heal in from two to three weeks, only rarely taking any considerably greater time. If, then, such an otitis should continue for three weeks in spite of lancing and wick drainage, the doctor is justified in proceeding as though he knew it was to run for an indefinite period.

As regards the treatment, everything depends upon what the cause may be that keeps up this discharge. Just as in a case of vaginal leucorrhea, you may cure the inflammation by local application, or be compelled to follow it into the cervix, urethra, endometrium, or possibly resort to tubal operations before discharge is stopped, so with the chronic running ear. In the first class of cases, those caused by inflammatory adenoid trouble or the persistence of discharge from nasal catarrh across the mouth of the Eustachian tube, it will be at once apparent that the underlying condition must be rectified. The adenoid gland, if enlarged or if diseased, no matter how small, must be thoroughly removed. All conditions of the nose keeping up a constant discharge, such as hypertrophy of the turbinates, deflection of the septum, sinus troubles, whether accompanied or not by polypi, should be properly remedied. After these various operations the doctor can with benefit paint the mouth of the Eustachian tube with 2% silver nitrate solution or use the Eustachian catheter and inject, after inflation, a little 2 per cent. camphor menthol, or both of these treatments. It will be found in a great many cases that the mouth of the tube is so sensitive to pressure of the tip that more can be done by simply painting with astringent preparations and using Politzer infiltration than by the use of the catheter. In connection with this treatment much benefit can be derived from certain antiseptics in the external ear; thus, however, only when the perforation of the drum membrane is of such size as to allow fair access to diseased parts. I would warn against the common practice of irrigating the canal of the ear with boric acid and other solutions. I believe that many a chronic running ear can be kept up by washings that would dry shortly under wick drainage.

It is comparatively rare, if a doctor is careful in his treatment, to find these simple cases of chronic catarrhal otitis that will not heal in a reasonable time. Unfortunately, however, it has been my experience that the overwhelming proportion of the cases are not so simple as this, that the disease has not only extended to the middle ear, but has involved the mastoid antrum and usually the entire mastoid cavity.

I think it is advisable to have an X-Ray examination made in every case of suspected chronicity. This if properly done, seldom fails to show a distinct blur over the region affected. A thoroughly competent skiagrapher is essential in this work. I will not attempt to quote statistics, for I believe that nothing less than several hundred observations from various operators can give a fair estimate, but, I think, that four out of five cases of chronic otitis will show under X-Ray distinct evidence of mastoid inflammation. In such cases, one may or may not uncover a history of a typical mastoid attack. You are all familiar with the time honored descriptions of acute mastoditis and recall the symptoms of pain on pressure over the mastoid antrum, fever, redness of skin, tenderness behind the ear, of auricle forced forward by the inflammation formed behind it, and all specialists at least are familiar with the symptom of sagging of the posterior superior canal wall. One must be on the alert, for perhaps only the last symptom may exist, and that to so slight a degree as to be noticed only by the trained aurist and usually only during an acute exacerbation of a chronic mastoiditis. The history may be extremely deceptive. Mastoid pain is frequently overlooked in the general headache or neuraliga. Perhaps the attending physician has not searched particularly to find the slight difference in tenderness over mastoid tip or antrum. I would emphasize the fact that just as in a large proportion of the cases sinus trouble which causes the chronic discharge from the nose, so in the chronic running ear a distinct mastoid inflammation may have existed for years without any of the ordinary symptoms named in the average text-book, excepting discharge. Pain over the mastoid is a symptom only of pressure, and if the cortex is thick and the pus has not worked through to the periosteum, absolutely no tenderness may be felt even on firm pressure. Redness and swelling of the skin is, of course, a symptom of external periostitis. In other words, the pus is already working through the external wall. Subjective pain is a symptom of pus under pressure. If this pus is formed in sufficient quantities to cause pressure upon the delicate nerve endings in the mastoid or middle ear, the patient may have severe pain. If, on the contrary, the drainage is free enough to carry off the pus as fast as it is formed, there may be no suffering whatever. On the other hand, pain persisting after proper lancing of the ear is very suggestive of mastoid trouble. It is not at all uncommon for perforation through the inner table of the skull to occur with so little pain that the patient does not recall it. I might also mention that very many physicians have made the mistake of diagnosing the external redness and swelling of a furuncular abscess as mastoiditis. An expert should not make this mistake, but no doctor who makes it a point to use X-Ray early will ever be caught napping in this fashion.

Where, then, we find a chronic running ear that is kept up by the discharge from an infected mastoid cavity, and I would say that these cases are the average ones that we see, it is futile to waste our time on ordinary treatment through the canal. These cases that run for months and even years are almost invariably chronic mastoid infections. If cured at all by nature, it is usually by the slow process of complete sclerosis of the mastoid cells. The doctor who waits for this waits also until all effective hearing is destroyed, to say nothing of running the very great chance of brain infection. Thes cases require surgery and it is a very great satisfaction not only to see the chronic discharge entirely disappear, but to find the hearing increased by from one hundred to one to two thousand per cent. after mastoid operation.

The treatment of infected cavities in any part of the body is the same—drainage. I believe that when that has thoroughly been effected most other work is likely to be meddlesome. When the middle ear has become the drip cup to an infected mastoid, it is foolish to drain only the drip cup, and laughable merely to clean away the leakage from this drip cup into the external ear. Drainage must be employed where the discharge can be drawn away harmlessly, instead of allowing it to ooze over the most delicate part of the whole conducting mechanism, the inner membrane. No one hesitates about cleaning out an abscess of the neck, why should he hesitate over one in the mastoid cells? The operation, done in time and by skilled hands, is practically devoid of danger. A slight scar and depression hidden behind the ear is practically nothing as against the increased thickening of the membrane and anchylosis of the stirrup bone, which means increasing and incurable deafness.

As to how much can be promised through operation in these cases, a little investigation is necessary. In the majority, perhaps, of these chronic running ears there are intermissions when the discharge entirely stops. If the hearing during the intermission is better than while the ear is running, one can confidently promise the patient that a simple mastoid will make permanent at least as good hearing as he had during the intermission. In the rarer cases, where discharge is continuous, a man must be guided by various tests by forks, but practically always improvement can be promised. The cases in which no improvement comes from simple operation are those where the ossicles have become anchylosed on account of the chronic inflammation extending around them. In such cases, perhaps more can be promised from radical operation.

The continuance of some discharge for a few weeks after a simple mastoid operation is rather the rule in cases where the ear has been running for a long time, i. e., for a year or more. This comes from involvement of the attic of the tympanum, which, of course, in simple operation is not touched, yet I emphatically protest against the too frequent use of a radical operation in these cases.

In examining specimens you will notice that the cells of the mastoid cavity are, as a rule, larger at the tip of the process than in other regions, also that each mastoid cell drains through a very narrow opening into the cavity of the antrum. Thus it takes but little inflammation to close the drainage channel of one or more cells, and we have incidentally the same train of symptoms which follows the swelling of the mucus membrane of the appendix. Pus must force its way through this narrow channel or cause necrosis of the bone. Necrosis will take the path of least resistance, and the pus may work out on the surface, forming first a periostitis and then an abscess behind the ear, or more frequently, it may burst through into the middle fossa of the skull, or possibly as abscess of the brain. If it occurs lower, the natural place for it to break will be over the lateral sinus, and we have therefore, first a subdural abscess, which if not properly drained, will cause clotting of the blood in the vein and this in turn, as soon as it becomes infected, will cause general pyaemia and death. This occurs more frequently in children and young adults. A perforation of the tip of the mastoid may occasionally occur and pus drain down outside of the skull and form a deep cervical abscess internal to the sterno-mastoid muscle.

It has been claimed by quite a number of authorities that it usually requires about two years for a chronic discharge to lead to necrosis of the bone in the mastoid or tympanic cavities. This depends entirely upon the nature of the germ. Where the infection is of streptococcic origin, necrosis may come in a very few days. Where it is of staphylococcic, the necrosis is very much slower, and it is still slower in the pneumococcic infections. Hence the prognosis must be gauged largely by bacteriological examination. There is no time to wait in pure streptococcus infection and not much where there is a considerable percentage of this germ.

One will have trouble in influencing his patients for their own good in the treatment of any deep seated conditions. Almost any person will permit the lancing of a superficial abscess, where he can see and feel the accumulation of pus. While he dreads even this slight operation, nevertheless he will have it done, as he can see the direct necessity of it. But when the abscess is inside of a bone where he feels only the pain, or, still worse, in cases where he notices only a flow of pus, so that his only annoyance is that of a chronic discharge, with so gradually increasing deafness as hardly to excite his apprehension, it may be quite difficult to secure the consent of the patient to the only means of cure. If one will always keep in his office a section of the temporal bone, so arranged as to show these cells; when one can point out to a patient that there is pus down in the tip of mastoid, that it must push its way through narrow channels the entire length of the mastoid process, through tiny openings into the antrum then past the narrow chink between the ossicles of the middle ear and the wall of the attic and drain directly over the most delicate point at the oval window, upon the integrity of which his hearing absolutely depends, and the membrane into the external ear, when one shows him that the use of medicine in the ear canal, with washings, etc., is essentially attempting to clean house by scrubbing out the vestibules without ever reaching even the halls, let alone the rooms of the house itself, the logic is rather conclusive and one does not have as great difficulty as before in securing his co-operation.

The wonder is often expressed that a little ear can run as long and hopelessly as it does. When one understands the anatomy thoroughly, the wonder is not that the ear runs so long, but that it ever stops. It is a great exhibition of the curative powers of nature to think that in certain cases of this kind nature does secure a complete subsidence of the inflammation and sometimes with a fair amount of hearing left. If the operation is done early, before anchylosis of the ossicles has occurred, or marked thickening of the membrane of the oval window and the rest of the tympanum, the chances of complete restoration to what we would call good business hearing are excellent.

In these days, there is but little excuse for allowing a chronic running ear to persist until the hearing is destroyed. It is inevitable that in disease of this sort there will be certain cases where but little, if any, improvement can be secured. These cases come under several heads. First, there are those where the discharge, either on account of its being markedly streptococcie or of long duration has led to considerable anchylosis and binding down of the ossicles by adhesions. In these cases no results needs be expected in the matter of hearing, through simple mastoid operation, although the discharge can usually be removed. In such a radical operation may be required, which consists, you all know after the simple operation, of removing the posterior wall of the ear canal, the external wall of the attic, the drum membrane with the malleus and incus, and curetting the entire tympanic cavity, excepting the round and oval window. This, carefully done, should stop all discharge, and, as a rule the hearing is at least as good as it was before.

Again, in other cases of chronic discharge, the necrosis may have gone inward into the petrous portion of the temporal bone and led to disease of the labyrinth, usually entirely destroying hearing and leading to serious danger of brain abscess. In these cases, again, nothing can be done in the way of relieving deafness and nothing but radical mastoid operation with careful treatment of the inner ear will cure. In case of chronic running ear where we find the canal of the ear occluded by polypi, we can practically always put it down for granted that there is disease of the ossicles. In these cases, as well, hearing has been very seriously and probably hopelessly impaired, and the simple mastoid will not, as a rule, prevent the further growth of polypi.

I have made no mention in this paper of the use of vaccines in the treatment of catarrhal discharge, for the reason that my experience has so far been inconclusive. I hope that the experience of others in this line will be brought out in discussion.

In conclusion, a brief summary:

Persistence of discharge from an ear treated by lancing and dry wick drainage is either, first, from continued infection and poor ventilation through the Eustachian tube, or second, continued infection from discharge from mastoid cells and attic.

X-Ray examination will settle the diagnosis. Early operation on throat or mastoid, or both, with proper after treatment will cure in the vast majority of cases. Ordinary washing and treatment with antiseptics in the canal is of itself of very little use.

Many patients will make light of our efforts in their behalf. A few years ago the many who advised operation for chronic appendicitis were laughed at. People really value acuteness of hearing highly. Danger to life is by no means slight in mastoid infections. Early operative treatment of the "Appendicitis of the Brain" is just as urgently required as in appendicitis of the abdomen, and we will soon be held accountable for not recognizing this condition and advising operation.

Healey Building

---

### IS SYPHILIS HEREDITARY?

E. Kilbourne Tullidge (Detroit Med. Journal: The majority of modern biologists disbelieve in the transmissibility of acquired characteristics in general, which include disease and deformity. Mammalian ova have not been shown to be phagocytci and Gartner has definitely shown that spermatozoa are not. Hohlff and Gartner have calvulated the number of spermatozoa in an ejaculation at over 26,000,000. (Note: The editor reported in the New York Medical Journal, June 4, 1910, three counts by hæmocytometer, respectively 346 million, 2,672 million and 286 million.) Owing, possible to missprints, we are unable to follow the mathematic argument as to the probability of true hereditary infection of the ovum, but it is extremely small. Colles' law as to the possibility of non-infection of the mother of a syphilitic child is no longer held to be true. Various experiments are cited as to the possibility of infection through the placenta, hence locating primarily in the liver of the fœtus, according with actual observation. Even if syphilis were hereditary, the mother would also be infected from the fœtus. —Medical Progress.

